# The influence of insurance status on waiting times in German acute care hospitals: an empirical analysis of new data

**DOI:** 10.1186/1475-9276-8-44

**Published:** 2009-12-21

**Authors:** Björn A Kuchinke, Dirk Sauerland, Ansgar Wübker

**Affiliations:** 1 Ernst-Abbe-Zentrum, Ehrenbergstraße 29, D-98693 Ilmenau, Germany; 2 Department of Institutional Economics and Health Systems Management, Witten/Herdecke University, Alfred-Herrhausen-Str 50, D-58448 Witten, Germany

## Abstract

**Background:**

There is an ongoing debate in Germany about the assumption that patients with private health insurance (PHI) benefit from better access to medical care, including shorter waiting times (Lüngen et al. 2008), compared to patients with statutory health insurance (SHI).

**Problem:**

Existing analyses of the determinants for waiting times in Germany are a) based on patient self-reports and b) do not cover the inpatient sector. This paper aims to fill both gaps by (i) generating new primary data and (ii) analyzing waiting times in German hospitals.

**Methods:**

We requested individual appointments from 485 hospitals within an experimental study design, allowing us to analyze the impact of PHI versus SHI on waiting times (Asplin et al. 2005).

**Results:**

In German acute care hospitals patients with PHI have significantly shorter waiting times than patients with SHI.

**Conclusion:**

Discrimination in waiting times by insurance status does occur in the German acute hospital sector. Since there is very little transparency in treatment quality in Germany, we do not know whether discrimination in waiting times leads to discrimination in the quality of treatment. This is an important issue for future research.

## Introduction and Objectives

In Germany, one of the main issues of the 2007 Health Reform was access to medical services. The legitimate political objective of securing access to medical services [1] is now basically ensured with the legally specified insurance for all citizens [2]. However, as there are still compulsorily- and privately-insured persons in Germany even after this reform, an important remaining question is whether access also depends on insurance type. In times of rationed medical supply, hospitals could implement waiting lists to discriminate between patients by urgency of treatment needs, but also by profitability. Holders of private health insurance (PHI) often have better access to more innovative and costly treatments than holders of statutory health insurance (SHI) [3]. Thus, discriminating between PHI and SHI patients is potentially profitable for providers of medical services. Indeed, empirical results reveal that private insurees benefit from better access to medical care including shorter waiting times. Studies using US data show that Medicaid patients were shown to have higher waiting times than privately insured patients, who pay more for equal treatments [4,5]. However empirical literature on the association between insurance status and waiting times in Germany is scarce.

Provision of services within the German health care system is divided between the ambulatory sector, including all office-based physicians, and the hospital sector. In the German ambulatory sector, where remuneration for treating PHI-patients is on average 20-35% higher than for treating SHI patients, the difference in waiting times between patient types amounts to 300% [6]. To-date there is no corollary study examining access to services in German acute care hospitals. Acute care hospitals are the most important care providers in Germany, and include all short-term hospitals with the facilities, medical staff and necessary personnel to diagnose, care and treat a wide range of acute conditions, including emergencies [7]. Therefore the objective of this paper is to determine empirically whether discrimination of patients by insurance status occurs within the German acute care hospitals.

We assume that the incentives for active discrimination in waiting times by insurance status are caused by differences in the insurance restrictions with which the service providers are confronted. For this reason, Section 2 will describe the relevant institutional background for health insurance in the German hospital sector. Section 3 presents the study design and describes the data. In Section 4 we describe the estimation strategy and present the results, which are then discussed in Section 5.

## The Role of Health Insurance in the German hospital sector

In 2004, German health authorities introduced a Prospective Payment System (PPS) for hospitals, creating strong incentives for economic discipline in the hospital sector. Under PPS hospitals get a fixed payment for each patient in a diagnosis-related group (DRG), regardless of the actual costs incurred for the care of the patient. A large literature documents hospitals' theoretical and empirical responses in introducing cost-saving or reimbursement-increasing measures under PPS. These measures include decreases in the length of stays [8], selective reductions in expenditures for high-cost patients [9], 'upcoding' - i.e. switching patients from appropriate lower-paying to higher-paying DRG in order to inflate reimbursement [10] and selection of profitable patients [11]. Due to health insurance regulation in Germany, patient insurance status is thus a potential indicator of profitability for hospitals. The main regulations are described below.

In Germany, PHI is only available to some segments of the population, namely civil-servants (compulsory PHI), the self-employed, and individuals with an annual income above 48.150 € (optional PHI). The rest of the population is covered by compulsory SHI [12]. The cost of holding a PHI depends on morbidity and age. Therefore, individuals with a lower morbidity profile tend to self-select into PHI while those with a higher morbidity profile choose SHI. Overall, due to compulsory coverage and self-selection, PHI holders are on average healthier and have higher incomes than SHI holders [13]. Moreover, the higher-income private insurees are more sensitive to long waiting times than SHI holders, as their opportunity cost of waiting (in terms of foregone income or leisure time) is higher [14]. This strong preference for low waiting times puts pressure on hospitals to lower their waiting times, in order not to lose private insurees to competing hospitals.

Moreover, the treatment of PHI holders can generate additional remuneration not available in basic SHI [15].^1 ^These are 'hotel-benefits' (private rooms), costlier treatment by the chief physician, and access to innovative and costly treatment methods. In 2006 this additional remuneration amounted to 2.5 billion €, or 4% of total hospital revenues. For a one-bed room only the extra revenue amounted to 82.61 Euro per day, or around 2.4% of average cost per patient in 2006 [16].

In sum, we have four arguments as to why (a) hospitals privilege the treatment of privately insured patients and (b) the waiting period is applied as a control instrument - to the advantage of the privately insured patient: the availability of compulsory PHI for higher income individuals; the possibility of self-selection into PHI for higher income (and lower morbidity) segments of the population; additional possibilities for income generation through the treatment of PHI patients; and the higher sensitivity of PHI patients to long waiting times. Therefore we formulate the following hypothesis:

*Hypothesis: In German acute care hospitals, private patients will get an appointment for treatment faster than compulsorily insured patients with the same medical indication*.

## Study Design and Data

### Study Design

Data on waiting times for appointments at acute care hospitals in Germany was not available prior to this study. Therefore, it was collected following an experimental study design, as proposed by Asplin et al. (2005). Trained graduate students, who posed as patients, called hospitals administrations using standard interview questions and data collection forms with the aim of obtaining an appointment for a physician consultation. During the call the simulated patients (callers) had to make clear that they had already been through a thorough medical check by an ambulatory doctor shortly before the appointment request, such that their diagnosis was already established. In Germany patients can only be admitted to a hospital either as an emergency case or by a referral of a general practitioner or a specialist. Usually, a general practitioner provides his patients with an appointment for a hospital stay. In order to directly obtain an appointment, our simulated patients told the hospital personnel that they had to abstain from a routine referral by their doctors due to a recent tenancy changeover to a new hometown. An important aspect of the experimental design of our study was to keep insurance status exogenous to waiting times. For this purpose, callers were told not to actively communicate their insurance type. Callers presented themselves as basic SHI holders only upon request by the personnel of the hospital.^2 ^Those hospitals actively requesting insurance type were then called a second time within a few days of the first call.^3 ^During this second call, the simulated patients claimed to be privately insured. Hospitals not asking for the insurance type were not called a second time. The callers collected information as to whether an appointment was consented to or refused, what the waiting time in days until the appointment was, and on which day of the week the call was made. Upon attainment, all appointments were cancelled in order not to bind hospital capacity. The calls were randomly distributed over weekdays (Monday to Friday) in 2007.

Before the interviews, a medical practitioner selected three clinical conditions for callers to use, based on two criteria. First, the conditions could not be life-threatening or considered emergency cases, but should necessitate a medical treatment within a short period (maximum two weeks) to minimize avoidable detrimental health effects. Second, the conditions should be treatable by hospitals within the sample of hospitals for which data on hospital characteristics was readily available. The chosen conditions are Weber B Fracture, cervical conization, and stenosis. Weber B Fracture is a fracture of the ankle joint treated operatively in surgical departments. Cervical conization is an operative treatment performed by gynecology departments when cancer is suspected. Stenosis is a constriction of the coronary vessels treated by a stent implantation in cardiology departments. The four-digit codes within the International Classification of Diagnoses in its 10^th^-German Modification are "S82.6" for Weber B Fracture, "I25.1" for stenosis and "C53.9" for cervical conisation.

### Basic Data and Sample Size

Hospitals in the study were selected on the basis of the Clinic Guide for Germany (Status 31.12.2003) [17]. Table 1 shows that of a total of 2,122 relevant hospitals, 1,339 have a surgical department, 235 hospitals have a cardiology department and 1,065 hospitals have a "gynaecology/obstetrics" department. The sample size is listed in table 1. ^4^

**Table 1:** Random check scope and executed calls

Plan hospitals with	Population	Calls with a successful appointment	Insurance asked for (called twice)	Exclusion
Surgery	1,339	194	19	39

Cardiology	235	107	58	30

Gynaecology	1,065	184	45	118

Total		485	122	187

Data collection took place from 04.25.2006 to 01.25.2007. In order to arrive at the desired sample size, more than 485 calls had to be made, as some of the randomly-selected hospitals only had general practitioners' wards in the required department or did not provide the desired service. The number of hospitals excluded is displayed as "exclusion" in the fifth column of Table 1. This indicates that, for example, for the surgical area 233 hospitals were called for the required 194 appointments.

The number of hospitals actively investigating the insurance status is displayed in Table 1, column 4. Out of a total of 485 hospitals, 122 actively investigated the insurance status. Thus 25% of the hospital departments called find the type of insurance relevant. To form a control group, these hospitals were called again in the second round. Thus a total of 607 appointments were made. Table 2 displays the variables generated.

**Table 2:** List of Variables

Variables evaluated for the entire sample	
**Name**	**Description**

Waiting period	Gross waiting period for an appointment

Waitingperiodqueried	Gross waiting period for an appointment at those hospitals querying the insurance status

Waitingperiod_n_queried	Gross waiting period for an appointment at those hospitals not querying the insurance status

Waitingperiodankle	Gross waiting period for an appointment for the diagnosis Weber B Fracture

Waitingperiodstenosis	Gross waiting period for an appointment for the diagnosis Stenosis

Waitingperiodconization	Gross waiting period for an appointment for the diagnosis Conisation

Waitingperiodprivhosp	Gross waiting period for an appointment at private hospitals

Waitingperiodpubhosp	Gross waiting period for an appointment at public hospitals

Waitingperiodcharhosp	Gross waiting period for an appointment at charitable hospitals

Privately Insured	1, if patient is privately insured, otherwise 0

Insurance Status Questioned	1, if hospital investigated actively the insurance status, otherwise 0

Weber B Fracture	1, if the diagnosis was "Weber B Fracture", otherwise 0

Stenosis	1, if the diagnosis was "Stenosis", otherwise 0

Cervical Conisation	1, if the diagnosis was "Cervical Conisation", otherwise 0

Privately Owned Hospital	1, if the hospital is privately owned, otherwise 0

Public Hospital	1, if the hospital is publicly owned, otherwise 0

Charitable Hospital	1, if the hospital is charity owned, otherwise 0

Number of beds	Number of beds in treating hospital

**Variables evaluated for the subgroup of hospitals investigating insurance status**	

**Name**	**Description**

Waitingperiodprivpat	Gross waiting period of privately insured patients with consideration only to those hospitals querying the insurance status

Waitingperiodcompat	Gross waiting period of compulsorily insured patients with consideration only to those hospitals querying the insurance status

Appointment1weekcompat	1, if patient is compulsorily insured and gets appointment within 1 week, otherwise 0

Appointment1weekprivpat	1, if patient is privately insured and gets appointment within 1 week, otherwise 0

Appointment2weekscompat	1, if patient is compulsorily insured and gets appointment within 2 weeks, otherwise 0

Appointment2weeksprivpat	1, if patient is privately insured and gets appointment within 2 weeks, otherwise 0

WaitingperiodcompatSt	Gross waiting period of compulsorily insured patients with the diagnosis "Stenosis", consideration only to those hospitals querying the insurance status

WaitingtimeprivpatSt	Gross waiting period of privately insured patients with the diagnosis "Stenosis", consideration only to those hospitals querying the insurance status

WaitingperiodcompatAnk	Gross waiting period of compulsorily insured patients with the diagnosis "Weber B Fracture", consideration only to those hospitals querying the insurance status

WaitingperiodprivpatAnk	Gross waiting period of privately insured patients with the diagnosis "Weber B Fracture", consideration only to those hospitals querying the insurance status

WaitingperiodprivpatCon	Gross waiting period of privately insured patients with the diagnosis "Cervical Conisation", consideration only to those hospitals querying the insurance status

WaitingperiodcompatCon	Gross waiting period of compulsorily insured patients with the diagnosis "Conisation", consideration only to those hospitals querying the insurance status

Table 3 presents descriptive statistics for the data. It displays the number of observations, the mean, the standard deviation and the minimum and maximum values of the data. All analyses were performed using Stata 9.0. In the upper portion of the table, statistics for the full sample are shown. The lower portion of the table presents the statistics for the subsample of hospitals actively requesting the insurance type (and therefore called twice). The upper part of table 3 shows that hospitals that investigated insurance type have on-average higher waiting times than those who did not. Further, the data reveal that average waiting times differ by clinical condition and hospital ownership. As the lower portion of the table shows, average waiting times clearly differ by insurance type, with PHI holders having shorter waiting times than SHI holders. The following statistical analyses will deliver more detailed insights regarding the impact of insurance status on waiting times.

**Table 3:** Descriptive Statistics

Variable	Observations	Mean	Standard deviation	Min	Max
Waiting period	607	8.3081	8.2857	1	56

Waitingperiodqueried	244	9.7541	7.6090	1	48

Waitingperiod_n_queried	363	6.4986	8.2238	1	56

Waitingperiodankle	213	2.5235	4.4204	1	54

Waitingperiodstenosis	165	13.3454	10.8844	1	56

Waitingperiodconization	229	8.7043	4.8478	1	36

Waitingperiodprivhosp	81	6.1358	8.3990	1	54

Waitingperiodpubhosp	294	7.8809	8.6623	1	56

Waitingperiodcharhosp	232	8.2974	7.2332	1	45

**Subsample of hospitals called twice**	**Observations**	**Mean**	**Standard deviation**	**Min**	**Max**

Waitingperiodprivpat	122	8.9667	7.0996	1	45

Waitingperiodcompat	122	10.5533	7.9788	1	48

Waitingperiodankle	38	3.3947	4.0706	1	23

Waitingperiodstenosis	116	12.8534	9.0518	1	48

Waitingperiodconization	90	8.4254	3.7148	1	21

Waitingperiodprivhosp	16	9.4375	7.4204	1	29

Waitingperiodpubhosp	130	9.0930	7.6663	1	48

Waitingperiodcharhosp	98	10.6667	7.5457	1	45

### Estimation Strategy

First we test our hypothesis using Ordinary Least Squares (OLS) regression. The logarithmic gross waiting period^5 ^is used as the dependent variable since gross waiting time is skewed left, indicated by maximum values strongly varying from the mean. This allows for an endogenous variable with approximate Gaussian distribution which is necessary for an undistorted estimator in the context of an OLS regression. The short form of the estimated equation is:

*β*_0 _stands for the estimate of the intercept, *I*_*i *_is a dummy for the insurance status of the patient; IAF_i _is a dummy indicating whether insurance status was actively investigated, D_i _is a vector of dummies indicating patient clinical conditions; X_h _is a vector of the other observed hospital characteristics listed in Table 2 and *u*_*ih *_is the error terms. We use specification tests (Ramsey Reset test for omitted variables; Breusch-Pagan/Cook-Weisberg test for heteroscedasticity) to check for violations of the OLS model assumptions. We also calculated the proportion of PHI and SHI patients who received an appointment within one week and within two weeks as a further key indicator of possible discrimination against SHI patients. As this comparison has dichotomous characteristics (appointment within one week equals 0, otherwise 1), we performed McNemar's Test. Finally, we conduct separate estimates for the subsamples of the three clinical conditions in order to check the robustness of the results.

## Results

### Overall Analysis

Table 4 presents the OLS results. Specification tests reveal that the OLS-model assumptions are not violated: A Reset test did not reject that the OLS-model has no omitted variable bias (F(3, 596) = 1.80; Prob > F = 0.1464) and a Breusch-Pagan/Cook-Weisberg test for heteroscedasticity could not reject homoskedastic errors (chi2(1) = 1.94; Prob > chi2 = 0.1639). The results show that PHI is related to shorter waiting times than SHI. Thus, insurance status is a significant predictor of waiting times. On average PHI-holders' wait was 1.6 days or 18.9% ([exact computation: 100(e^(-0.21)^-1)]) shorter for treatment than the subgroup of SHI-holders who were actively questioned about insurance status. Further, we find that this subgroup of SHI-holders has to wait 21% (([exact computation: 100(e^(0.19)^-1)]) longer for treatment than the group of SHI-holders who were not questioned about their insurance status (control group). This hints at overall lower capacity utilization in these hospitals as compared to those which actively investigated the insurance type of the patients.

**Table 4:** Determinants of Waiting Time and Questioning

Variable	Gross waiting time (logarithm)	Questioned
	**OLS (for all diagnosis)**	**Probit (for all diagnosis)**

Privately Insured	-0.21 (0.089)**	

Insurance Status Questioned	0.191 (0.078)**	

Weber B Fracture	-1.52 (0.678)***	-0.229 (0.045)***

Stenosis	0.221 (0.075)***	0.312 (0.051)***

Privately Owned Hospital	-0.179 (0.091)**	-0.200 (0.060)**

Public Hospital	-0.138 (0.063)**	0.016 (0.466)

Number of Beds	0.139 (0.084)	-0.360 (0.663)

Intercept	2.011 (0.069)***	

		

Observations	607	607

F-Value/LR Chi2	103.99	

Prob > F/Prob > Chi2	0.0000	0.0000

Adj. R^2 ^or Pseudo^1 ^R^2^**	0.5762	0.1490

^1 ^Pseudo R^2 ^cannot be compared to adj. R^2 ^because of the different methods of calculation used. Standard errors are shown in brackets. Level of significance: ***P < 0.01, **P < 0.05.

As a by-product of these empirical analyses, we also found significant associations of both the clinical diagnoses and hospital ownership with waiting time. With regard to diagnosis group, we find that patients diagnosed with Weber B Fracture receive an appointment significantly faster than patients diagnosed with Cervical Conisation (control group). Concretely patients with Weber B Fracture have on average a 78% ([exact computation: 100(e^(1.52)^-1)]) shorter waiting time than patients diagnosed with cancer. The group with Cervical Conisation, however, received an appointment significantly faster (24.6%; [exact computation: 100(e^(0.22)^-1)]) than patients diagnosed with Stenosis.

Regarding hospital ownership, we find that patients of public and privately owned hospitals receive appointments significantly faster than patients of charitable hospitals. Patients of public hospitals receive an appointment 12.9% [exact computation: 100(e^(-0.138)^-1)] faster than patients of charitable hospitals. Appointments at private hospitals are 16.4% [exact computation: 100(e^(-0.179)^-1)] faster than at charitable hospitals. The probit estimate (left column) also shows a positive relationship between waiting time for treatment and diagnosis-specific questioning behavior. In addition to being the group with the shortest waiting time for treatment, patients with Weber B Fracture also show the lowest probability of being asked to state their insurance status. Further, the probit estimate shows that privately-owned hospitals ask about insurance status significantly less often than public or charitable hospitals.

There are also significant differences in the proportions of those patients who receive an appointment within one week: while 41% of privately-insured patients receive an appointment within one week, this is the case for only 28% of compulsorily insured patients. This result does not change significantly if the time period to the appointment is extended. Only 73% of the compulsorily-insured patients received an appointment within two weeks of the call, while the proportion for privately-insured patients is 81%. Both of these differences - for the period of one week as well as for two weeks - are statistically significant, as shown by the results in Table 5.

**Table 5:** Proportion of patients with an appointment for surgery after 1 or 2 weeks respectively

Variable	Observations	Mean	McNemar's chi2	P-value
Appointment1weekcompat	122	0.28	6.40	0.0114**
		
Appointment1weekprivpat	122	0.41		

Appointment2weekscompat	122	0.73	3.13	0.071*
		
Appointment2weeksprivpat	122	0.81		

Standard error in brackets, significance level: ***P < 0.01, **P < 0.05, *P < 0.10.

As a result we may thus state:

*The zero hypothesis cannot be disproven based on the data we have collected. We have to assume that (a) a part of the hospitals in Germany actively apply waiting periods as an instrument to control their patient flow and that (b) private patients will get faster access to in-patient medical services than compulsory insured patients*.

### Detailed Analysis

Table 6 documents the results of the detailed analysis for each of the three clinical conditions. Because of the considerably lower number of observations, a number of estimation coefficients are no longer significant. However, it can be said that insurance status within each observed diagnosis groups shows the expected effect. In addition, the other estimation coefficients confirm our fundamental analysis. Only the estimator of privately funded hospitals for stenosis is not in accordance with the core analysis. Furthermore, a significant negative correlation with waiting time can be shown for the Weber B Fracture and stenosis diagnoses for the privately owned hospitals as compared to the control group, hospitals owned by charitable organizations, in spite of the relatively small sample size.

**Table 6:** Diagnoses

Variable	Gross Waiting Time (logarithm)		
	**Tobit^1 ^(Weber B Fracture)**	**OLS (Stenosis)**	**OLS (Conisation)**

Privately Insured	-0.362 (0.191)**	-0.152 (0.148)	-0.084 (0.107)

Insurance Status Questioned	0.535 (0.138)***	0.148 (0.156)	0.008 (0.088)

Privately Owned Hospital	-0.231 (0.127)*	0.174 (0.236)	-0.227 (0.116)*

Public Hospital	-0.223 (0.098)**	-0.093 (0.141)	-0.116 (0.076)

Number of Beds	0.075 (0.129)	0.519 (0.205)**	0.239 (0.119)**

Intercept	-0.093 (0.087)	1.981(0.175)***	1.995(0.076)***

			

Observations	68 uncensored/144 censored	165	230

F-Value/LR Chi2	20.67	1.33	3.06

Prob > F/Prob > Chi2	0.0021	0.2489	0.0068

Adj. R^2 ^or Pseudo R^2 ^(Mc Fadden)	0.0473	0.0120	0.0512

Standard error in Brackets. Level of Significance: ***P < 0.01, **P < 0.05, *P < 0.10.

## Conclusion

The objective of this paper was to assess the impact of private and statutory health insurance on waiting times in German acute care hospitals. For this purpose, we collected individual appointments from hospitals within an experimental study design [4]. We find that 25% of German hospitals actively query insurance status. In these hospitals the PHI-holders wait almost 1.6 days or 18.9% less for an appointment for treatment than SHI-holders. Only 28% of SHI-holders get an appointment within one week, compared to 41% of private insurees. Even if the observation period is extended to two weeks, PHI patients are significantly more likely than SHI patients to receive an appointment. As such, we have to assume that some hospitals in Germany actively use waiting times as an instrument to control their patient flow and that PHI-holders therefore have faster access to inpatient medical services than SHI-holders. The preferential treatment of PHI-holders may be explainable by profit and costs considerations. In Germany hospitals treating PHI-holders profit from remuneration generated by additional services, which are often unavailable to SHI-holders. Further, due to health insurance regulation PHI-holders are on average a relative healthy high income group, which is at lower risk of generating unremunerated costs for the hospital. Furthermore, PHI-holders are more sensitive to long waiting times than SHI-holders. In sum, privileging PHI-holders with shorter waiting times potentially ensures hospitals a greater number of more profitable patients.

As a by-product of our empirical analyses we find two additional interesting relationships. First, waiting times differ for the different clinical diagnoses. We find that patients diagnosed with Weber B Fracture receive an appointment significantly faster than patients diagnosed with cervical consisation or stenosis. Shorter waiting times in the case of Weber B Fractures are most likely related to the occurrence of acute pain for these patients. Another explanation of shorter waiting times in the case of Weber B Fracture may be that the DRG-remuneration plays an important role in hospitals' determination of waiting time. The DRG-benchmark index for the treatment of an Weber B fracture - the diagnosis with the lowest waiting time - is 2.15, followed by stenosis (benchmark index 0.96) and conisation (benchmark index 0.488), implying the highest revenues for treating Weber B fractures [18]. However, our data do not allow us to assess the impact of the analysed clinical diagnoses in terms of "good DRG" or "bad DRG". In order to give a more detailed judgment of the impact of financial incentives on waiting times, this topic should be considered in future empirical work. Second, we find that privately owned hospitals do have significantly shorter waiting times than charitable hospitals. As there is an ongoing process of privatization and reorganization of the German hospital market, a closer look at the impact of ownership on waiting times may also be a fruitful research topic.^6^

Our study is limited in several respects. The economic context raises the question of why only 25 percent of the called hospitals actively investigated the insurance status - and 75 percent did not. One possible answer may be that the capacities in these hospitals are not utilised to the extent that would make a selection expedient, so that higher utilisation may initially take priority. An underutilised capacity may also explain why the average waiting time in the non-investigating hospitals is shorter than in the investigating hospitals (cf. table 3). Capacity may be a more severe restriction for the latter, and would motivate a more intense selection of patients. However, the data we collected did not enable the exploration of these possible relationships. Further, we do not focus on the SHI-group with voluntary supplementary private insurance covering additional hospital services (cf. footnote 1). As this exclusion could bias our results, this group should be examined by further research.

The medical context of this study raises concerns regarding whether and how differences in waiting times might affect health status. Considering the magnitude of the effects, it is not possible to say whether an additional 2 days of waiting time for SHI patients may damage their health. However, as most waiting times are within a medically acceptable timeframe, most likely it should not.

Another limitation concerns the generalisability of our results: Our callers were - by definition - posing as new patients at every clinic they contacted. As typically patients obtain appointments by referral through the ambulatory health sector in Germany, we cannot rule out that the difference in waiting times between PHI and SHI patients is influenced systematically by our study design. However this does not negate the importance of our findings. First, national [6] and international studies [4] confirm the influence of financial incentives on waiting times. Second, even though patients typically obtain appointments by referral through the ambulatory sector, it is not unusual for patients to try to get an appointment over the telephone without seeking in-person care first. Further regarding generalisability, we acknowlege that our study included only three diagnoses. Therefore we do not know if differences in waiting times between SHI and PHI would significantly change given other diagnoses. Furthermore, for these diagnoses it is quite atypical for the patient themselves to make a clinical appointment. Additional insights could be gained by examining strictly elective surgical procedures, such as total hip replacement or cholecystectomy.

While we clearly show that discrimination in waiting times by insurance status does occur in the German acute hospital sector, we do not know whether discrimination in waiting times carries on to discrimination in the treatment quality. This is an important issue, especially as there is very little transparency in quality of treatment in Germany. In the end, patients cannot judge which hospitals provide the best treatment. As long as quality remains nontransparent, it will be easier for hospitals to discriminate amongst patients by insurance status. This is an important issue for future research.

## Competing interests

The authors declare that they have no competing interests.

## Authors' contributions

BAK collected the data, participated in the design of the study and drafted the manuscript. DS participated in the design and coordination of the study and drafted the manuscript. AW participated in the design of the study, drafted the manuscript and performed the statistical analysis. All authors read and approved the final manuscript.

## Appendix

^1 ^SHI holders can voluntarily purchase supplementary private insurance to cover additional hospital services. Currently 5.1 Million (7.1%) of SHI-Insurees chose this option [15].

^2 ^We do not focus on the SHI-group with voluntary supplementary private insurance covering additional hospital services (cf. footnote 1) and discuss this non-consideration in the limitations section.

^3 ^The average lag between first and second call was 7 days. The lag was meant to be kept short in order to minimize the risk that a sudden change in a hospital's capacity utilization could significantly impact waiting times. Where possible, in order to prevent the interviewer from being re-identified by the personnel of the hospital, second calls were done by different callers. Furthermore, caller identification for all outgoing calls was blocked.

^4 ^For calculation purposes, we applied the minimum required random check scopes with a safety probability of 95%, a maximum tolerated random check error of 7%, and a proportion of hospitals for reasons of missing experience data conservatively to 50%.

^5 ^The gross waiting time determined in the study contains the number of days from the day of the call to the appointment, including all public holidays and weekends (Saturday/Sunday).

^6 ^Furthermore we neglected the impact of market structure on waiting times. Since the market structure has an impact on treatment alternatives on site, this topic should be considered by further research either [19,20].
